# Towards a more robust comparative oncology: a Bayesian reanalysis of Peto’s paradox and discussion of comparative cancer risk studies in vertebrates

**DOI:** 10.1098/rsos.250840

**Published:** 2025-07-09

**Authors:** Antoine M. Dujon, Peter A. Biro, Beata Ujvari, Frédéric Thomas

**Affiliations:** ^1^ School of Life and Environmental Sciences, Deakin University, Waurn Ponds, Victoria 3216, Australia; ^2^ CREEC/(CREES), MIVEGEC, Unité Mixte de Recherches, IRD 224–CNRS 5290, Université de Montpellier, Montpellier, France

**Keywords:** cancer, phylogenie, evolutionary mismatch, zoo animals, cancer risk, scientific method

## Abstract

The multistage carcinogenesis model predicts that cancer risk should increase with body size and longevity owing to greater cell numbers and divisions, which provide more opportunities for mutations. However, the perceived lack of such associations across species, named ‘Peto’s paradox’, suggests that larger or longer-lived animals may have evolved enhanced cancer suppression mechanisms. Empirical tests of this paradox have been limited by data availability, but large-scale zoo datasets now enable comparative analyses of cancer prevalence in vertebrates. Currently used statistical methods, however, often fail to adequately account for uncertainty in key model parameters. In this study, we use Bayesian methods to reanalyse these datasets and explore Peto’s paradox, emphasizing the importance of quantifying uncertainty in comparative oncology. Our results show that body mass is positively associated with malignancy risk in mammals and amphibians, while it is negatively associated with cancer mortality in mammals. Longevity is positively associated with malignancy risk in non-avian sauropsids and amphibians. However, these relationships are accompanied by effect sizes with substantial uncertainty, primarily owing to small sample sizes. Through simulations, we demonstrate the limitations of current datasets and models. We also discuss the broader implications of Peto’s paradox and suggest recommendations for improving future research on cancer risk across species.

## 1. Introduction

Cancer has been present since the emergence of multicellular life [1–3]. Because malignant cells can compromise host survival, species have evolved diverse anticancer defences [4–7]. These defences are energetically costly and involve trade-offs with other vital functions, so cancer can still arise under certain conditions [8–11]. While our understanding of why cancer susceptibility varies across species is improving [12–15], important questions remain [11]. One key example, emblematic of the field is Peto’s paradox [15,16]: why do not larger, longer-lived species, despite having more cells and longer time frames for mutations, exhibit higher cancer rates, as predicted by the multistage carcinogenesis theory [16,17]? Given its significance and the ongoing debates, it provokes, Peto’s paradox provides a compelling example for examining how methodological choices in statistical analyses influence our understanding of the relationship between cancer risk factors and prevalence across species. It also offers a useful lens through which to evaluate how effect sizes are estimated and how uncertainty and limitations are addressed in comparative oncology studies.

**Figure 1.**
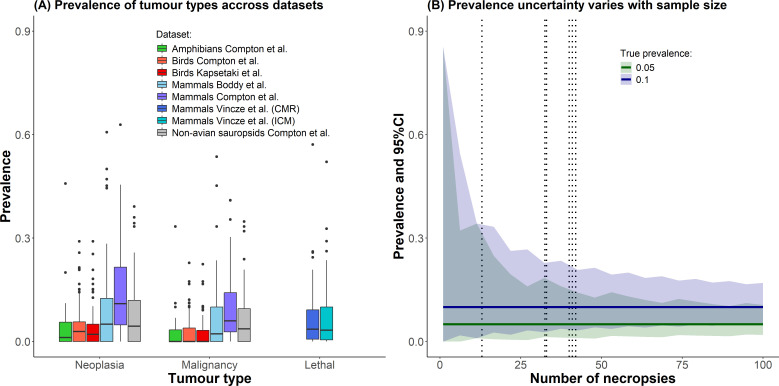
(A) Distribution of measured tumour prevalence (neoplasia, malignant and lethal) across all datasets included in this study. In most datasets, observed prevalence is relatively low. (B) Effect of sample size (number of necropsies) on the width of the 95% credible intervals (CIs) for cancer prevalence estimates. Solid lines represent true prevalence values, while shaded error bands indicate CIs. Dotted vertical lines mark the median number of necropsies per dataset. The figure illustrates that uncertainty in prevalence estimates remains substantial in over half of the data prevalence measurement, primarily owing to limited sample sizes.

Peto’s paradox is a well-explored topic with extensive literature [18–22]. The multistage carcinogenesis theory, which underpins the paradox, successfully predicts cancer risk in species like humans and dogs [23–25], where cancer incidence increases with body size and longevity, in proportions that are expected from the model’s predictions [16]. However, these trends are absent or only weakly observed when comparing neoplasia, malignancy or cancer mortality data across different species [26,27]. Various mechanistic explanations have been proposed to address the paradox, with adaptation and anticancer defence mechanisms that are often significantly different between species. For instance, elephants and Xenarthra exhibit duplication of tumour suppressor genes [28,29], while rodent species over 10 kg show repression of somatic telomerase activity and replicative senescence [15]. Bats have robust immune systems [30], and naked mole rats produce protective molecules such as hyaluronic acid or exhibit hypersensitivity to contact inhibition [31,32]. This list is not exhaustive and other potential mechanisms also exist (see the following reviews [14,15,33]). Fundamentally, these mechanisms suggest that evolution has been selecting for anticancer defences in long-lived and large animals to prevent fitness-damaging tumours [18,34]. However, despite the substantial amount of research, most studies have involved relatively few species, and the testing of Peto’s paradox across a broader range of species has only recently become feasible [35] owing to rapid release of new datasets. Those new datasets of captive animals compiled from multiple zoos on a global scale have increasingly facilitated comparative analyses which have explored the relationships between body mass, longevity and cancer prevalence or mortality across various species ([26,27,36–40]; figure 1A). For this purpose, those studies used phylogenetic regression models to estimate the average slope between body mass and/or longevity (most often log-transformed) and the prevalence of neoplasia, malignancy and lethal tumours. It is by using the value of those slopes and their significance that the ecologists develop their ecological interpretation of the datasets. So far, these analyses suggest that body mass and longevity are poor predictors of cancer risk. When significant the modelled average prevalence increases only marginally, by a few per cent across the full observed range of body sizes and lifespans, and the proportion of variance explained by the models (marginal *R*^²^) remains relatively low. This limited predictive power could stem from several factors: the datasets may be too small or noisy to detect real effects (leading to false negatives); the use of captive data may unintentionally bias results towards species more prone to developing neoplasia, malignancy, or lethal cancers (leading to false positives); or there may genuinely be no effect of body mass and longevity on cancer risk, though such a conclusion requires strong confidence in the absence of an association. Thus, to be robust, comparative analyses must accurately quantify effect sizes, characterize the associated uncertainties and present them in a way that clearly conveys both magnitude and variability. Currently, the heterogeneity in methods used to link cancer risk to prevalence makes it challenging to compare effect sizes and their associated uncertainties across comparative oncology studies.

Comparative analyses linking cancer prevalence to risk factors use linear models that incorporate phylogenetic information to account for species' shared evolutionary history [41–43] to test broad patterns across species. Such analyses are often conducted using frequentist methods (e.g. phylogenetic generalized least square models [41]), using the point estimate of prevalence as a response variable, an approach which face challenges in computing uncertainty around key model estimates. Indeed, the data are binomial (presence or absence of tumour) and so should be analysed assuming a binomial probability distribution and assuming binomial errors. With binomial data, the uncertainty of a prevalence estimate increases with the observed proportion to a maximum at *p* = 0.5 and uncertainty increases as the number of observations in that group declines (e.g. the number of necropsies for a species, see figure 1B). In currently used datasets, small sample sizes make it difficult to distinguish a true prevalence of 0.1 from zero, with sample sizes under 25 yielding very wide credible intervals (figure 1B). Importantly, this uncertainty will further interact with the phylogeny during modelling, creating additional uncertainty [41,42].

Currently, the effect of the variability in the number of necropsied individuals used to determine the prevalence for each species on the effect sizes of interest has not been fully explored. By using a Bayesian approach to the familiar binomial regression, it becomes possible to use posterior predicted values to estimate quantities of interest along with their credible intervals (e.g. to display error bands which are currently rarely represented in comparative oncology studies and important tool for data visualization); it also permits post hoc tests and hypothesis testing in a framework that offers more flexibility compared to frequentist approaches [44]. As further datasets are made available, Bayesian models will allow meta-analyses to be conducted with repeated measurement per species while accounting for heterogeneity in data sources and the phylogenies between species (see for example a recent example on animal behaviour that used the *brms* R package [45]). We thus argue that using Bayesian phylogenetic regression models will be crucial for testing hypotheses about the factors that potentially influence cancer defences in animals especially since those effect sizes are likely to be small and estimated from sparse data (discussed above). We therefore provide additional context and details commonly used in Bayesian phylogenetic regression models, aimed at supporting researchers, particularly those in comparative oncology, who may wish to apply these methods.

In this study, we thus use Peto’s paradox as a case study and present an alternative Bayesian method to the frequentist approaches commonly used in recent comparative studies on cancer risk across species. We reanalyse multiple distinct datasets with varying degrees of biological overlap, selected based on two key criteria: (i) they were originally published and openly shared by the study authors, allowing their reanalysis; and (ii) they provide an opportunity to both demonstrate our analytical framework and conduct a more methodologically robust evaluation of Peto’s paradox. We also investigate the effects of the variation in the number of necropsies across species on how reliable the slope between a cancer risk factor and cancer prevalence can be. This approach can be continuously refined as more datasets become available, providing a valuable tool for future research but can also be applied to any potential cancer risk factor of interest.

## 2. Methods

### 2.1. Datasets description

We obtained a total of seven datasets, three for mammals, two for birds, one for amphibians and one for non-avian sauropsids (here limited to squamates, turtles and crocodiles), from four different published sources. Below, we summarize the key characteristics of these datasets and encourage readers to consult the original publications for complete details (refer to table 1 for sample sizes and summary statistics, and the electronic supplementary material, S1 for an overlap in the number of species between datasets). These datasets provide a snapshot of how the authors who conducted those comparative studies linking cancer risk to body mass or longevity prepared and organized their data. Reanalysing these data using more robust methods thus allows us to assess whether the same trends persist or if different patterns emerge. Additionally, we describe the modifications (if any) made to the datasets for the purposes of this study. We did not include the dataset from Bulls *et al.* [38] in our study, as the raw data was not made available with their preprint at the time of writing this manuscript.

**Table 1.** Summary statistics of the various dataset used in this study (ICM,cumulative incidence of cancer mortality; CMR, cumulative mortality risk).

body mass (kg)					
dataset	type	min	median	max	number of species
Vincze *et al*. [26] (ICM)	mammals	0.018	17.75	1499.455	172
Vincze *et al*. [26] (CMR)	mammals	0.018	13.03	1499.455	191
Compton *et al*. [27]	mammals	0.00718	6.95	3178	102
Boddy *et al*. [37]	mammals	0.05	10.25	4800	37
Compton *et al*. [27]	birds	0.0095	0.2145	23	106
Kapsetaki *et al*. [39]	birds	0.0095	0.2145	23	104
Compton *et al*. [27]	non-avian sauropsids	0.01	0.4965	51.5	42
Compton *et al*. [27]	amphibians	0.00035	0.0457	0.534	30

maximum longevity (month)					
Vincze *et al*. [26] (ICM)	mammals	43.2	264	734.4	167
Vincze *et al*. [26] (CMR)	mammals	43.2	267.6	816	186
Compton *et al*. [27]	mammals	45.6	255	786	98
Boddy *et al*. [37]	mammals	54	260.4	780	37
Compton *et al*. [27]	birds	70	270	789.6	59
Kapsetaki *et al*. [39]	birds	6	255.6	789.6	66
Compton *et al*. [27]	non-avian sauropsids	15.6	234	521.76	49
Compton *et al*. [27]	amphibians	48	129.6	480	30

The dataset provided by Vincze *et al.* [26] documents cancer-related mortalities in animals, specifically cases where anticancer defences failed, and cancer was identified as a primary cause of death. By focusing on lethal cancer outcomes, this dataset provides an opportunity to examine potential associations between lethal cancer susceptibility and life-history traits such as body size and longevity. The dataset includes two prevalence measurements in mammals: cumulative mortality risk, which is the ratio of animals with cancer that was considered to have significantly contributed to animal death to the number of necropsied individuals across 191 species, and the cumulative incidence of cancer mortality, calculated using Kaplan–Meier estimates that account for the censoring of animals during data collection across 172 species. The data is aggregated from Species360 and the Zoological Information Management System, an international non-profit organization that maintains a centralized, real-time database of animals in human care, compiling information from over 1200 zoos worldwide. Cancer is only recorded in the dataset curated by Vinzce *et al.* for deceased animals and only if the veterinary pathologist conducting the postmortem examination deemed it a contributing factor to the animal’s death. Only species with postmortem pathological records available for at least 20 adult individuals, regardless of the cause of death, were included in their analyses (with a median of 40 and a range of 20−413 necropsies per species). Vincze *et al*. also excluded species that were domesticated, along with their wild ancestors, owing to the widely recognized link between domestication, inbreeding depression and an increased cancer risk [46]. The dataset provides body mass estimates but does not include maximum longevity estimates; instead, it offers the average number of days lived after reaching sexual maturity. To maintain consistency in maximum longevity data across studies, we obtained estimates for 186 species from the AnAge database ([47]; https://genomics.senescence.info).

The dataset provided by Boddy *et al.* [37] includes neoplasia and malignancy prevalence data for 37 mammal species. This data is an aggregate of prevalence rates reported by Griner [48] and additional data collected from mammals housed at the San Diego Zoo and San Diego Zoo Safari Park. Complete post-mortem examinations and histopathology were conducted on animals from the San Diego Zoo, while histopathology was performed on cases compiled by Griner. The median number of necropsied individuals per species is 13 with a range of 4 to 91. The dataset also includes body mass and maximum longevity estimates.

The dataset provided by Compton *et al.* [27] includes neoplasia and malignancy prevalence data for mammals, birds, sauropsids and amphibians. The data was aggregated from 99 zoological institutions, aquariums and other facilities that care for animals under managed conditions. It also includes the individuals from Boddy *et al.* [37] and Griner [48]. Neoplasia and malignancies were histologically identified by board-certified veterinary pathologists, and cases without histological examination of suspected neoplasms were excluded. Neonatal records were also excluded to avoid bias from the high mortality rates commonly seen in neonates and infants across many species. All species have a minimum of 20 necropsies. The dataset includes body mass and maximum longevity estimates for most bird, mammal and non-avian sauropsids species; but this data is lacking for most amphibians which we thus obtained from Stark *et al.* [49]. Additionally, one species, the Indian peafowl (*Pavo cristatus*), was reclassified as a bird rather than a reptile as originally listed. Four non-avian sauropsid species were excluded from the analysis because they were not present in the phylogenetic tree used. In this dataset, the median number of necropsies was 41 (range: 20−354) for mammals, 32.5 (range: 20−477) for birds and 33 (range: 20−96) for non-avian sauropsids and 42 (range: 20−304) for amphibians.

The dataset provided by Kapsetaki *et al.* [39] includes neoplasia and malignancy prevalence data for 108 bird species, sourced from necropsies performed at the Association of Zoos and Aquariums institutions and is a subset of Compton *et al.* [27] with differences in the number of species and individuals included. For this dataset, necropsies were conducted by veterinarians or veterinary pathologists and involved a thorough examination of each organ system, with samples from each organ or any identified abnormalities submitted for histopathological analysis. The median number of necropsied per species was 32.5, with a range of 20−477. The authors excluded cancers in subadult individuals. The dataset includes body mass and maximum longevity estimates, and we supplemented it with maximum longevity data for eight additional species from the AnAge database.

For simplicity, we will refer in the result section to the datasets using the name of the first author of each publication, while fully acknowledging the contributions of all co-authors.

### 2.2. Statistical analyses

#### 2.2.1. Methods used in previous studies

Previous studies have used various phylogenetic regression models to analyse cancer prevalence datasets in vertebrates in relation to their body size and life expectancy. For instance, Compton *et al.* [27] and Boddy *et al.* [37] used Gaussian phylogenetic regression models which used the raw point estimate of the prevalences as a response variable while testing for different models of evolution (e.g. Brownian motion or Ornstein–Uhlenbeck). Kapsetaki *et al.* [39] arcsine-square-root transformed the prevalence point estimates to meet the assumption of normality of the response variable before fitting Gaussian models. In Compton *et al.* [27] and Kapsetaki *et al.* [39], the prevalence measurements were also weighted by 1/(square root of the number of necropsies per species) to account for differences in the number of necropsied individuals across species.

Owing to the presence of a large number of zeros in the data, some comparative analyses of mammal cancer risk sometimes used a two-step approach, resembling a zero-inflated Gaussian model. Studies [26,50,51] first applied binomial phylogenetic regressions to model the probability of malignant cancer detection in a species. For species with malignant cancer, they then used Gaussian phylogenetic regressions on logit-transformed prevalences, weighting measurements by 1/log(necropsy count) to account for sample size variation to predict how the prevalence scales in relation to different risk factors.

A limitation of the frequentist methods described above is the difficulty in obtaining confidence intervals and quantifying uncertainty for certain model parameters. For example, it is not always straightforward to plot average prevalence regression lines along with their associated 95% confidence interval bands to visualize the data. It is also not always straightforward to interpret a slope estimate when the response variable is based on certain types of transformations (e.g. arsine or arcsine-square-root transformed). Using a binomial regression model addresses the binomial nature of the data directly, and with the usual logit link function to linearize the model and determine odds ratios (OR) which are easier to interpret [52].

#### 2.2.2. Bayesian phylogenetic regression models

In this publication, we use the flexibility of Bayesian statistics to reanalyse the aforementioned datasets by modelling animal counts directly using a binomial distribution. This approach estimates the prevalence of neoplasia, malignant or lethal tumours by directly modelling the number of successes (a necropsied animal with tumour) and failures (a necropsied animal without tumour) instead of weighting prevalence estimates by the number of necropsies. The flexibility of the R *brms* package allows us to account for evolutionary history and build models with different link functions and residual correlation structures to account for the effect of the phylogeny. In addition, Bayesian models generate posterior distributions for each parameter of interest, allowing for the calculation of credible intervals, which facilitates visualizing the uncertainty around these effects [53]. Credible intervals, typically set at 95%, are calculated from the 2.5 and 97.5% percentiles of a posterior distribution but can extend to other ranges (e.g. 50%, 75%). Quantifying this uncertainty in effect sizes is important for understanding their ecological relevance.

Recent comparative oncology studies frequently conduct subgroup analyses, either to test specific biological hypotheses within particular taxonomic groups or to address limitations in data availability (e.g. [26,39,50,51,54,55]). Following this established approach, we performed separate binomial Bayesian phylogenetic regression analyses with logit link functions [53] for each major taxonomic group (mammals, non-avian sauropsids, birds and amphibians) within each dataset. These models predicted the proportion of individuals within a species that developed a specific tumour type (neoplasia, malignant or lethal; see table 1). Body mass (in kg) and maximum life expectancy (in months) were included as log10-transformed fixed effects to account for potential allometric effects. The models estimate the average impact of a variable on tumour prevalence across species [56,57]. To account for the relatedness between species and the lack of statistical independence, the models also included a variance–covariance matrix constraining the residuals of the model based on a phylogenetic tree and a model of evolution [42].

#### 2.2.3. Calculation of the variance–covariance matrix to account for the phylogeny

To compute the variance–covariance matrices used in the models, we used a consensus phylogenetic tree from various sources. For mammals, we used the consensus tree derived from a set of 1000 equally plausible trees provided by [58]. For birds, we created a consensus tree from a set of 1000 trees available on the Birdtree.org website [59], using the *consensus.edges* function from the *phytools* R package [60]. Similarly, for non-avian sauropsid, we generated a consensus tree from a set of 1000 trees provided by [59]. For amphibians, we used the consensus tree provided by [49,61].

The choice of the evolution model used to calculate the variance–covariance matrices constraining the residual variance in phylogenetic regression models can have an impact on the estimation of effect sizes and their associated uncertainty [42]. Compton *et al.* [27] and Kapsetaki *et al.* [39] recently suggested that an Ornstein–Uhlenbeck process is a potential good model to describe the evolution of cancer risk across phylogenies. However, it has also been argued that Ornstein–Uhlenbeck based models can be challenging to estimate when sample sizes, such as the number of species, are relatively small ([62,63] but see also [64]). To address this, we compared, for each risk factor, a model using a variance–covariance matrix derived from an Ornstein–Uhlenbeck process with one where the strength of the phylogenetic signal was estimated using Pagel’s method of scaling the internal branches of a phylogenetic tree (the λ parameter). We evaluated model performance using both the leave-one-out information criterion (LOOIC) and the widely applicable information criterion (WAIC), see [65].

Out of the 56 model comparisons, the models incorporating a variance–covariance matrix based on an Ornstein–Uhlenbeck process outperformed those based on Pagel’s method of scaling in 49 cases (WAIC differences were often greater than 2; see the electronic supplementary material, S2 for model comparisons and effect size estimates for all models). In the stances where Pagel’s method of scaling performed better, the differences were minimal, with LOOIC and WAIC differences of less than 1. We therefore used an Ornstein–Uhlenbeck model of evolution to fit all subsequent models in this study.

#### 2.2.4. Model checks and validation

Compared to frequentist models, Bayesian models require a couple of additional steps to ensure that the models are appropriately fitted and parameters correctly estimated. All Bayesian models were run for a minimum of 5000 iterations, with a burn-in period of 2000 iterations and a thinning interval of 5, using four chains. We applied default uninformative priors for all models. To ensure that the model converged appropriately, we used the following criteria: we ensured that autocorrelation between iterations within each chain was low (*R* < 0.1). We verified that the Rhat values for each estimated parameter were equal to 1, and that both the bulk effective sample size and the tail effective sample size were greater than 1000. We visually inspected the posterior distributions of each parameter to confirm they were unimodal and performed posterior predictive checks to identify any models with a poor fit [53,66]. Residual distribution of the models was inspected using the DHARMa package [67]. To interpret the slope coefficients of model that successfully converged, we converted them to ORs and calculated their associated 95% credible intervals [52].

Since species are not phylogenetically independent, and thus there is a lack of statistical independence between each measurement, plotting raw data points can be misleading, so alternative visualization methods must be used [68]. For example, the average regression line calculated with a mixed effect model (which includes phylogenetic regression models) may not be located in the middle of a point cloud where the eye would tend to place it as it would do for a simple linear regression model [69]. Thus, for each model, we plotted the marginal effects of body mass and life expectancy on cancer risk [70]. Marginal effects were calculated by holding all parameters constant except the variable of interest, allowing us to visualize its impact on average cancer risk across species. Here, we focused on visualizing the average effects of body mass and maximum life expectancy, along with their error bands while accounting for phylogeny and repeated measurements in meta-analysis models. To visualize uncertainty, we calculated 95% Bayesian credible intervals using Jeffrey’s prior, suited for species with 40 necropsies or less [71] and overlaid them with model predictions. All prevalence estimates reported in this study include these intervals.

#### 2.2.5. Examining the impact of variability in necropsy numbers across species

We conducted simulations to examine how different necropsy sample sizes and species' phylogenies affect the estimated effect size linking a risk factor to cancer prevalence (e.g. OR). Sensitivity analyses are crucial because cancer prevalence is typically low, with small differences in effect sizes and average prevalence across the range of maximum longevity and body mass [26,27,38,39]. This is important to consider because uncertainty can be very large in small sample size situations with binomial data (figure 1A). In each simulation, the observed prevalence in the dataset (i.e. the proportion of animals with neoplasia or malignancy provided in each dataset) was held constant and the number of necropsies per species (sample size) was altered. In those simulations, we randomly shuffled the observed sample sizes across species within each dataset, adjusting the neoplasia or malignancy counts to preserve the initial prevalence ratios. We ran each simulation 50 times to balance time and number of combinations, as Bayesian phylogenetic regression models are time-intensive to fit. Given the uneven necropsy distribution (most species had low counts), we used this approach to check if model trends stayed consistent with different sampling, while preserving observed prevalence estimates. If species sampling is generally appropriate, simulations should return consistent results. Effect size estimates would be consistent in their trend direction (positive or negative) and significance (with 95% credible intervals not overlapping zero). This would suggest that, although the association’s magnitude may be uncertain, its direction would be reliable. Inconsistent results would indicate that variability in sample sizes across species impacts the estimates, reducing confidence in the trend’s direction. It is also important to note that these simulations are not designed to capture all potential sources of noise and bias in the dataset, but rather to highlight a specific factor, here the effect of necropsy sample size. Other factors should be addressed in future studies.

#### 2.2.6. Statistical software

All statistical analyses were conducted using R v. 4.3.3 [72] and the RStudio graphical user interface [73]. Phylogenetic trees were handled with the ape package [74]. The annotated R scripts and the raw datasets used in the analyses are available on GitHub (https://github.com/adujon/PetoVertebrates).

## 3. Results

**Figure 2.**
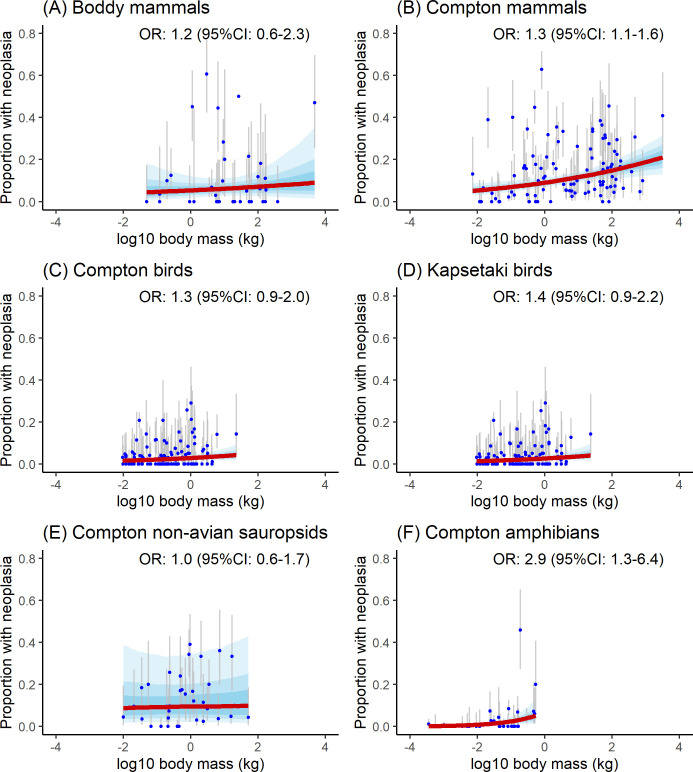
Marginal effect of body mass (log_10_ in kg) on neoplasia prevalence: (A, B) mammals, (C, D) birds, (E) non-avian sauropsids, and (F) amphibians. Solid red lines show the marginal effect; blue bands indicate 50, 75 and 95% credible intervals (CIs). Blue dots represent prevalence measurements with grey bars for 95% CIs.

**Figure 3.**
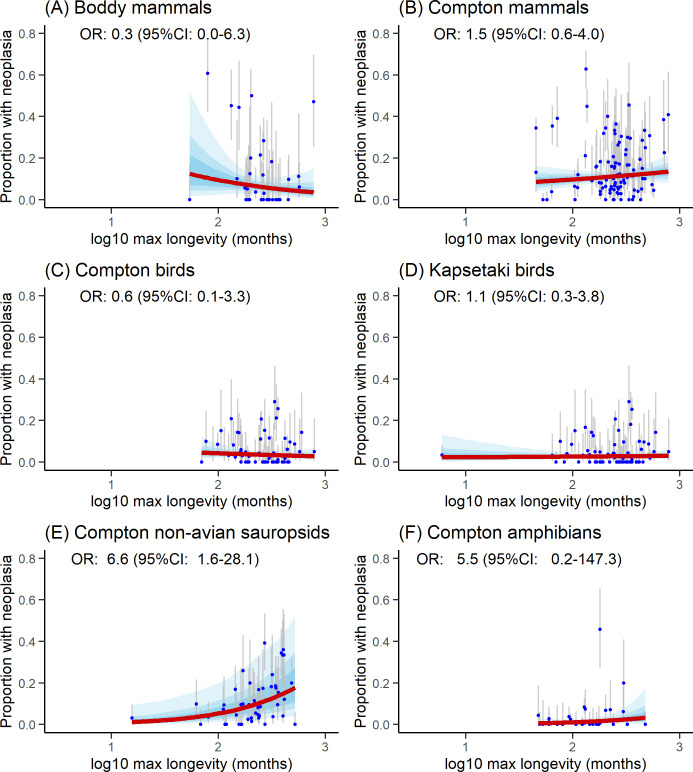
Marginal effect of maximum longevity (log_10_ in months) on neoplasia prevalence: (A, B) mammals, (C, D) birds, (E) non-avian sauropsids, and (G) amphibians. Solid red lines show the marginal effect; blue bands indicate 50, 75 and 95% credible intervals (CIs). Blue dots represent prevalence measurements with grey bars for 95% CIs.

**Figure 4.**
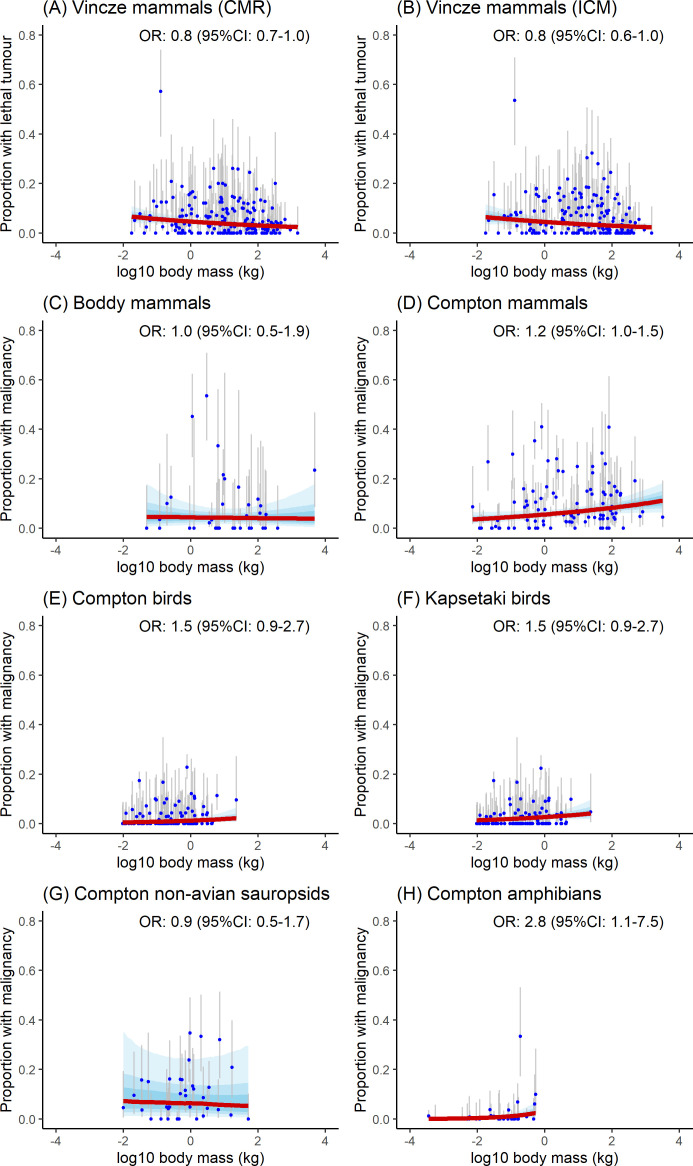
Marginal effect of body mass (log_10_ in kg) on malignancy and lethal tumour prevalence: (A–D) mammals, (E, F) birds and (G) non-avian sauropsids, and (H) amphibians. Solid red lines show the marginal effect; blue bands indicate 50, 75 and 95% credible intervals (CIs). Blue dots represent prevalence measurements with grey bars for 95% CIs.

**Figure 5.**
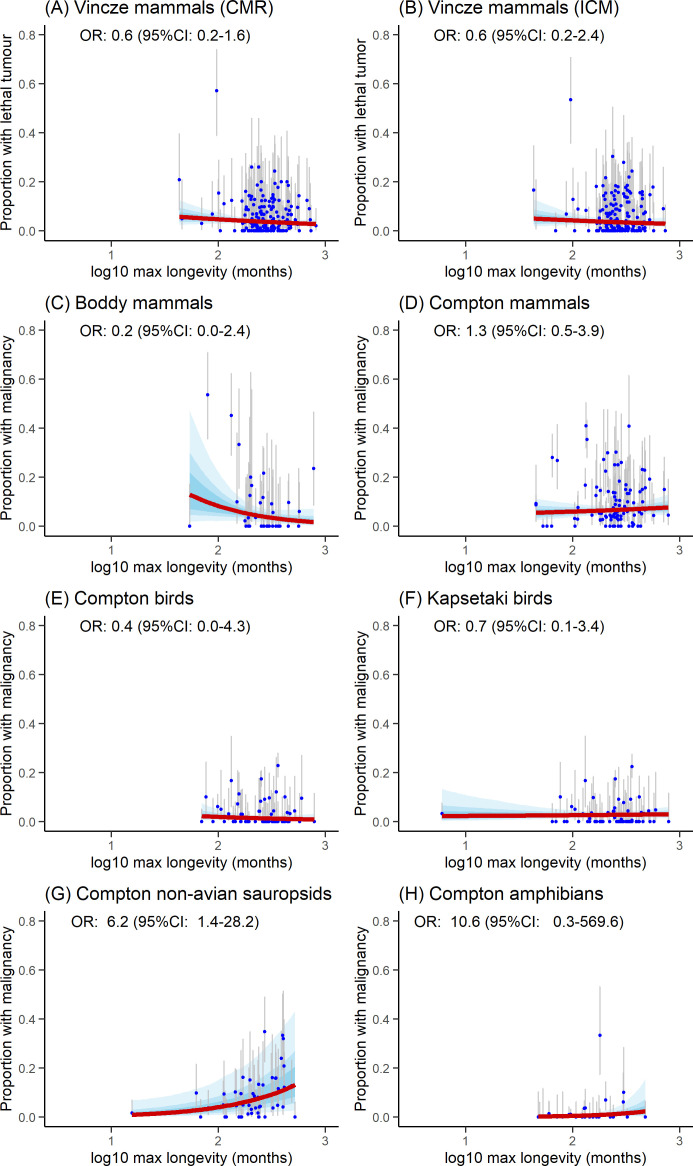
Marginal effect of maximum longevity (log_10_ in months) on malignancy and lethal tumour prevalence: (A–D) mammals, (E, F) birds, (G) non-avian sauropsids, and (H) amphibians. Solid red lines show the marginal effect; blue bands indicate 50, 75 and 95% credible intervals (CIs). Blue dots represent prevalence measurements with grey bars for 95% CIs.

Detailed OR (with their 95% credible intervals) for each model and dataset are provided in figures 2–5.

### 3.1. Neoplasia prevalence

**Figure 6.**
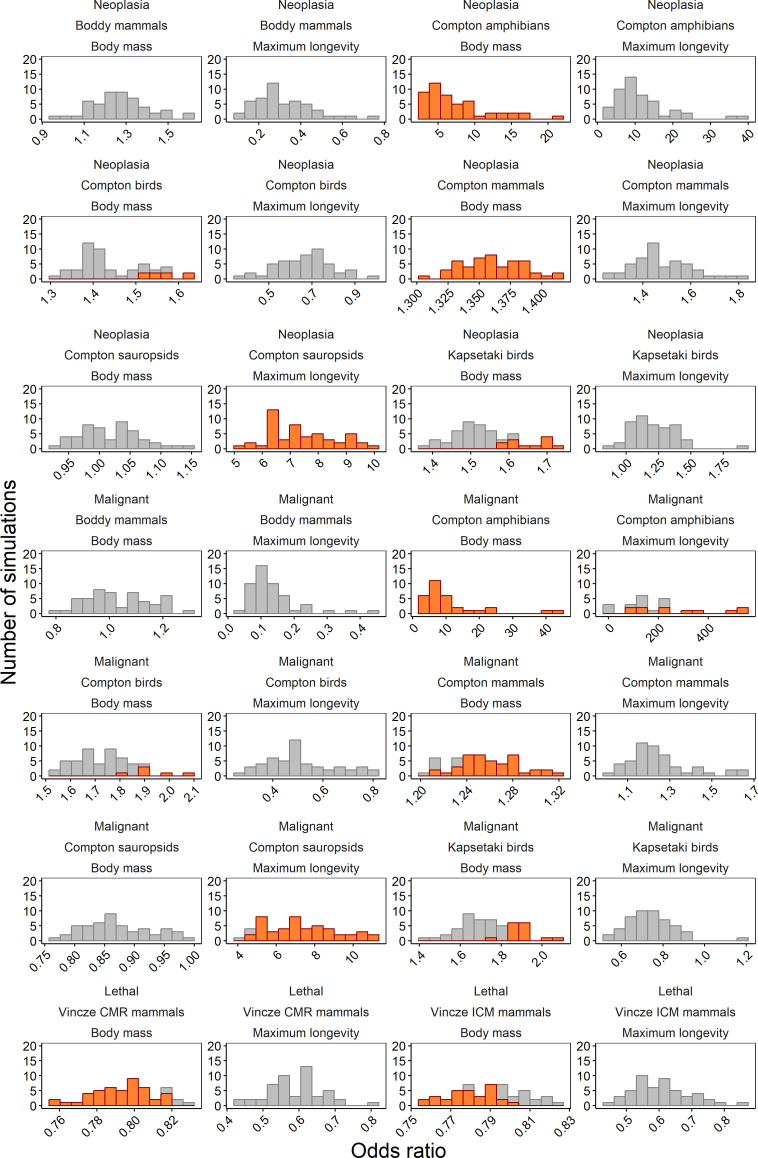
Results of the re-sampling simulations demonstrate the effect of keeping the observed prevalence of neoplasia or malignancies and lethal tumours constant while varying the number of necropsies (sample size) per species (detailed in ‘Methods’). The grey bars represent simulations where the odds ratios were not significant, while the orange bars indicate simulations where the odds ratios were significant.

In mammals, neoplasia prevalence showed a positive association with body mass in Compton’s dataset (unlike in Boddy’s dataset; figure 2A,B), a trend also observed in amphibians. However, no significant association was found in non-avian sauropsids or birds (figure 2C–E). Resampling simulations supported these patterns: body mass and neoplasia prevalence were consistently and significantly correlated in Compton’s mammal dataset (narrow effect size range; figure 6) and in amphibians (but with a broad effect size range). By contrast, simulations for Boddy’s mammal dataset and Compton’s non-avian sauropsids yielded non-significant associations. Results for birds were more variable, with 16% (8 out of 50) of simulations showing a significant positive association in Compton’s dataset and 24% (12 out of 50) in Kapsetaki’s dataset, while the remainder were non-significant.

Neoplasia prevalence was also positively associated with maximum longevity in Compton’s non-avian sauropsids, but no significant relationships were detected in other datasets (figure 3). Resampling simulations confirmed a consistent positive association for non-avian sauropsids, albeit with substantial variation in OR (figure 6). For all other datasets, simulations showed wide effect size variability and no significant association with longevity.

When neoplasia prevalence was modelled using both body mass and maximum longevity as predictors, body mass retained a significant effect in Compton’s mammal and amphibian datasets, while longevity remained significant in non-avian sauropsids. For all other taxa, neither predictor showed a significant effect (see the electronic supplementary material, S2). After including an interaction term between body mass and maximum longevity, the only variable that remained significant was the effect of body mass on neoplasia prevalence in non-avian sauropsids.

### 3.2. Malignancy prevalence

Our analyses revealed distinct patterns in the relationship between malignancy prevalence and body mass across taxonomic groups. A positive association was observed in Compton’s mammal dataset and amphibians, while no significant association was detected in non-avian sauropsids, birds or Boddy’s mammal dataset. Resampling simulations demonstrated these patterns should be nuanced (figure 6): Compton’s mammal dataset showed significant positive associations in 84% of simulations (42 out of 50), with amphibians displaying consistently significant positive associations in all simulations but with a large variation in effect sizes (indicative of a lack of robustness). Bird datasets exhibited mixed results, with significant positive associations in 16% (8 out of 50) and 24% (12 out of 50) of simulations for Compton’s and Kapsetaki’s datasets, respectively. No associations were found in simulations for Boddy’s mammal dataset or Compton’s non-avian sauropsids.

Regarding longevity, Compton’s non-avian sauropsid dataset and amphibians showed a positive association between malignancy prevalence and maximum longevity (figure 5). This finding was robust in resampling simulations, with 94% (47 out of 50) showing significant positive associations (figure 6). The simulation for amphibians showed the trend observed for amphibians is unreliable with extremely large variations in effect size when the number of necropsies is altered. All other datasets consistently showed a lack of significant associations.

When malignancy prevalence was modelled as a function of both body mass and maximum longevity, body mass had a marginally significant effect in Compton’s mammal dataset and a significant effect in amphibians, while longevity was significant for non-avian sauropsids. All other models became non-significant when an interaction term between body mass and longevity was added with the exception of non-avian sauropsids, where body mass retained its significance (see the electronic supplementary material, S2).

### 3.3. Mortality prevalence

Reanalysis of Vincze’s cancer mortality mammal dataset revealed a small negative association between body mass and both cancer mortality risk and incidence of cancer mortality, while no association was detected with maximum longevity (figure 4). Resampling simulations showed significant negative associations with body mass in 90% of simulations (45 out of 50) for cumulative mortality risk and 60% (30 out of 50) for the incidence of cancer mortality, with the remaining simulations showing non-significant results highlighting important uncertainty in those trends. By contrast, all simulations returned non-significant associations between the two measures of cancer mortality and maximum longevity. The negative trend between body mass and cancer lethality persisted for both prevalence measurement types when body mass and maximum longevity were both included predictors, but this effect disappeared when an interaction term between the two variables was introduced (electronic supplementary material, S2).

The observed variability in the trend estimates for all types of tumours highlights the importance of sampling differences among datasets. Particularly, the number of necropsies per species emerges as a crucial factor influencing estimates of the relationships between body mass, malignancy prevalence and mortality prevalence across species.

## 4. Discussion

In this study, we aimed to assess how uncertainty in existing comparative oncology datasets influences the observed relationship between cancer risk factors and neoplasia, malignancy and mortality prevalence. We reanalysed published datasets using a Bayesian framework to evaluate Peto’s paradox and better understand the uncertainty in average prevalence estimates. Our analyses showed a weak positive association between body mass and neoplasia or malignancies in mammals and amphibians, as well as between maximum longevity and neoplasia in non-avian sauropsids and amphibians, based on Compton’s dataset. These trends were observed in previous studies, and it is reassuring that they persist when more robust methods are used. However, our re-sampling simulations indicate substantial uncertainty regarding their true effect sizes. Additionally, using the whole Vincze’s dataset, we identified a weak and significant negative association between cancer mortality incidence and body mass in mammals. This finding contrasts with the absence of a significant trend reported in Vincze’s original publication. The lack of a clear association between longevity and cancer prevalence (to the exemption of non-avian sauropsids and a very noisy trend for amphibians), despite the multistage carcinogenesis model predicting higher cancer risk in longer-lived species [16], provides some support for Peto’s paradox but the available evidence obtained from the reanalysed datasets remains insufficient to conclusively confirm or reject the paradox, for reasons outlined below.

Differences in trends between neoplasia, malignancy and cancer-related mortality can be attributed to several factors. Neoplasia and malignancies in which positive trends are observed with body size are not always lethal, and animals may die from other causes before the cancer becomes fatal. As animals age, the selective pressure for cancer suppression is expected to weaken [10], allowing tumours to develop that are not necessarily life-threatening. In their dataset, Compton *et al.* [27] observed that most neoplasia are observed in animals that died before the average species lifespan. This suggests that most neoplasia never become malignant and impact animal health, or alternatively that elevated rates of neoplasia may reflect evolutionary mismatches in captive environments increasing the probability that cells develop neoplastic characteristics above the levels that cancer suppression systems evolved for. This contrasts with patterns observed when considering only cancers that significantly contributed to mortality, cases that indicate a breakdown of anticancer defences and in which a negative trend is observed. Several factors may explain the observed discrepancies observed when reanalysing Vincze’s dataset: (i) our analytical approach differed by using the complete dataset rather than fitting a model limited to species in which cancer was detected (as in Vincze’s method) and using a binomial model rather than a Gaussian one; (ii) larger species may exhibit greater resistance to lethal cancers, potentially requiring tumours to reach larger sizes before causing mortality; and (iii) larger captive animals might receive superior care, reducing cancer mortality probability compared to smaller species. Regardless of which hypothesis is correct, our resampling simulations also indicate that sampling variability in necropsy frequency across species substantially influences all analyses.

Several limitations, which are shared by all recently published comparative studies investigating cancer prevalence in vertebrates, affect our conclusions. First, neoplasia, malignancies and mortality prevalences were calculated by aggregating multiple cancer types, with variations in their respective prevalences [75–77] and future data may show that the positive trend between body mass and cancer risk across species does not hold when examining specific cancer types. For example, the number of driver mutations required to initiate a cancer varies between species and cancer types with any additional driver mutation reducing the probability of cancer initiation (e.g. 1−10 in humans [78] and an estimated four in dogs [25]). This variation in the number of driver mutations considerably impacts the prevalences predicted by a multistage carcinogenesis model for each cancer type [16]. There are also large variations in the number of necropsied individuals between species, and the uncertainty around prevalence estimates is large for species with a low number of necropsies which is reflected by our re-sampling simulations especially when using body mass as a risk factor. Should the number of necropsies across species have been sampled differently, some of the observed trends could have had different directions and significance levels and interpreted in terms of evidence supporting or against Peto’s paradox. Thus, the current necropsy sampling regime is introducing substantial noise in the datasets and does not currently allow us to have a definitive answer on whether cancer risk is associated with body mass. In addition, while the available data on cancer in captive animals is among the best currently available, it may not accurately represent cancer trends in wild species or the oncogenic pressures they face in their natural environments and for which they evolved cancer suppression mechanisms [12,79]. It is possible the observed trends are the results from evolutionary mismatches owing to captivity, which differs in terms of nutrition, exposure to pollution, parasites and physical activity levels from the natural environments where these species developed their anticancer defences [13,50] and that indeed in those altered conditions Peto’s paradox could be partially refuted owing to the increased and artificial exposure to oncogenic risks. In addition, the datasets are still limited, for example, when compared to the total of approximately 6400 extant mammals [80] or approximately 18 000 bird species [81]. Similarly, the trend observed between longevity and cancer risk in non-avian sauropsids and amphibians requires to be confirmed with additional studies. As demonstrated with Boddy’s mammalian dataset, which shows a consistent lack of association between maximum longevity, body mass and cancer prevalence trends may shift as the range of species included in the analysis increases.

A crucial step in fully testing and understanding Peto’s paradox will be to accurately assess the discrepancy between cancer risk predictions based on body mass (e.g. using a multistage carcinogenesis model), cell numbers or cell division numbers [82,83] and the actual observed cancer risk (see [24,25] for examples in humans and dogs). This requires several conditions: reasonably estimating the number of cells across species, determining the number of lifetime somatic cell divisions per species, estimating the probability of a somatic mutation per cell division, and estimating the number of driver mutations required to initiate a cancer of a given type. While mutation rates in mammals inversely scale with longevity, the mutation probability per division remains challenging to quantify accurately [84]. If the obtention of such data is achieved, we will very likely find that larger species experience less cancer than predicted by cell count and cell division numbers alone. It is the magnitude of this difference between predicted and observed prevalence that is of key interest, because it will help our understanding of how large species might trade off cancer protection for energy allocated to reproduction and fitness [8,11,51,85]. However, the current uncertainty surrounding prevalence measurements in the available datasets and the other raised limitations poses a significant challenge to this analysis and the paradox is still primarily unresolved (contrary to what is claimed in [40]) in a satisfactory way.

Our study highlights the importance of collecting more data on cancer risk across species to enhance statistical methods and reduce effect size prediction uncertainty. It is not uncommon in ecology to observe a decline effect: following the reporting of an initial statistically significant trend, further data is collected to study it and as more analyses are conducted the effect sizes associated with this trend decrease to become non-significant (e.g. [86,87]). Thus, both significant and non-significant effect sizes should be reported in future studies and interpreted in an evolutionary context to avoid publication bias in the field [9,12,88,89]. Developing easy-to-use tools for easier sensitivity and power analyses will be an important step to determine the minimum effect sizes a dataset can detect. Further modelling should also explore how neoplasia or malignancy prevalence differences impact species' evolutionary trajectories in their ecological contexts including for small prevalences [11]. For example, for prey species, predators might prevent the evolution of additional anticancer defences if these defences increase predation risk [90,91]. Similarly, intense intraspecific competition is predicted to lead to higher cancer incidences [85]. In addition, quantifying the cost of tumour burden in large animals will be an important step to understand a potential association between body mass and cancer especially if the trend is weak. At equal proportion of their body mass a tumour in a large animal may not exert the same cost on the host as for a smaller one. Malignant tumours may be disadvantaged in larger hosts, for example through the formation of hypertumours [22,92]. It is also important to note that while comparative analyses can show associations, they rely on observational data and may not be sufficient in establishing causality between risk factors and cancer prevalence in vertebrates. A key future challenge lies in analysing observational datasets to test causal relationships and identify potential confounding variables. While methods such as confirmatory path analysis (e.g. [93]) are specifically designed for this purpose, they may still fall short of uncovering the true underlying patterns in the data [94].

In this study, we intentionally focused our Bayesian analyses on the effects of body mass and maximum longevity on cancer risk in vertebrates, as these factors are central to Peto’s paradox. However, species exist within a cancer risk landscape [95,96] where multiple risk factors interact at different spatio-temporal scales and the logic underlying the paradox can be extended to other risk factors especially if species are exposed to it over many generations [97]. Comparative analyses have explored various cancer risk factors, such as diet, placentation, litter size, gestation length and parasite removal in zoos [26,27,39,50,98]. Future studies should include these variables to explain observed trends. For example, across mammalian species, longer gestation periods are associated with larger body mass, reduced neoplasia and malignancy prevalence and lower cancer mortality risk [37,51,99]. Established risk factors should be also included as confounding variables, as it would be with alcohol and tobacco in human cancer research. For example, diet type, particularly in species that consume other vertebrates, consistently shows an increased cancer risk in multiple studies [26,50,51].

While our Bayesian analyses generally replicate existing findings, they also reveal an important limitation in prior work: a lack of attention to the uncertainty surrounding reported trends. Bayesian models offer a distinct advantage by explicitly incorporating uncertainty at all levels, phylogenetic structure, parameter estimates and latent variables, making them especially valuable in evolutionary biology, where data are often sparse and noisy. Our results demonstrate that some previously reported associations are statistically fragile when tested using more rigorous phylogenetic methods and simulations. Rather than discrediting earlier studies, we recognize their valuable contributions while urging caution in interpreting their findings. By highlighting these limitations, we aim to encourage a more careful and nuanced approach to understanding cancer risk across species and to avoid premature conclusions that may not hold up to further scrutiny. As with many ecological questions [88,100], the initial concepts behind Peto’s paradox will evolve into a more nuanced understanding of how body mass and longevity influence cancer risk. For this, robust statistical tools are needed to understand complex ecological datasets. As more resources are made available (e.g. [101,102]) scientists should thus familiarize themselves with those valuable tools to conduct comparative studies to meet the key challenges in the field of ecology and evolution of cancer.

## Data Availability

All datasets used in this publication were obtained from previous studies. The codes used to produce the analyses in this manuscript are available at [103]. Supplementary material is available online [104].
